# Preconditioning by Hydrogen Peroxide Enhances Multiple Properties of Human *Decidua Basalis* Mesenchymal Stem/Multipotent Stromal Cells

**DOI:** 10.1155/2018/6480793

**Published:** 2018-04-29

**Authors:** T. Khatlani, D. Algudiri, R. Alenzi, A. M. Al Subayyil, F. M. Abomaray, E. Bahattab, A. S. AlAskar, B. Kalionis, M. F. El-Muzaini, M. H. Abumaree

**Affiliations:** ^1^Stem Cells and Regenerative Medicine Department, King Abdullah International Medical Research Center, King Abdulaziz Medical City, Ministry of National Guard Health Affairs, Mail Code 1515, P.O. Box 22490, Riyadh 11426, Saudi Arabia; ^2^Department of Clinical Science, Intervention and Technology, Division of Obstetrics and Gynecology, Karolinska Institutet, 14186 Stockholm, Sweden; ^3^Center for Hematology and Regenerative Medicine, Karolinska Institutet, 14186 Stockholm, Sweden; ^4^National Center for Stem Cell Technology, Life Sciences and Environment Research Institute, King Abdulaziz City for Science and Technology, P.O. Box 6086, Riyadh 11442, Saudi Arabia; ^5^College of Medicine, King Saud Bin Abdulaziz University for Health Sciences, King Abdulaziz Medical City, Ministry of National Guard Health Affairs, Mail Code 3124, P.O. Box 3660, Riyadh 11481, Saudi Arabia; ^6^Adult Hematology and Stem Cell Transplantation, King Abdulaziz Medical City, Ministry of National Guard Health Affairs, Mail Code 1515, P.O. Box 22490, Riyadh 11426, Saudi Arabia; ^7^Department of Obstetrics and Gynaecology and Department of Maternal-Fetal Medicine Pregnancy Research Centre, Royal Women's Hospital, University of Melbourne, Parkville, VIC 3052, Australia; ^8^Department of Obstetrics and Gynaecology, King Abdulaziz Medical City, Ministry of National Guard Health Affairs, Mail Code 3124, P.O. Box 3660, Riyadh 11481, Saudi Arabia; ^9^College of Science and Health Professions, King Saud bin Abdulaziz University for Health Sciences, King Abdulaziz Medical City, Ministry of National Guard Health Affairs, Mail Code 3124, P.O. Box 3660, Riyadh 11481, Saudi Arabia

## Abstract

Stem cell-based therapies rely on stem cell ability to repair in an oxidative stress environment. Preconditioning of mesenchymal stem cells (MSCs) to a stress environment has beneficial effects on their ability to repair injured tissues. We previously reported that MSCs from the *decidua basalis* (DBMSCs) of human placenta have many important cellular functions that make them potentially useful for cell-based therapies. Here, we studied the effect of DBMSC preconditioning to a stress environment. DBMSCs were exposed to various concentrations of hydrogen peroxide (H_2_O_2_), and their functions were then assessed. DBMSC expression of immune molecules after preconditioning was also determined. DBMSC preconditioning with H_2_O_2_ enhanced their proliferation, colonogenicity, adhesion, and migration. In addition, DBMSCs regardless of H_2_O_2_ treatment displayed antiangiogenic activity. H_2_O_2_ preconditioning also increased DBMSC expression of genes that promote cellular functions and decreased the expression of genes, which have opposite effect on their functions. Preconditioning also reduced DBMSC expression of IL-1*β*, but had no effects on the expression of other immune molecules that promote proliferation, adhesion, and migration. These data show that DBMSCs resist a toxic environment, which adds to their potential as a candidate stem cell type for treating various diseases in hostile environments.

## 1. Introduction

MSCs are isolated from many human adult tissues, such as placenta [1, 2]. MSCs have multipotent differentiation potential, which is important for tissue regeneration [3], and have immunosuppressive properties [4–7]. These characteristics make MSCs an attractive cell source for cell-based therapies. However, the use of MSCs in cell therapy is hindered by several important limitations. For example, the isolation and expansion of MSCs *in vitro* are associated with oxidative stress that reduces their proliferation and differentiation potentials, life span, immunomodulatory properties, and stemness [8]. In this study, we focus on oxidative stress, which results from an imbalance between prooxidant molecules including reactive oxygen and nitrogen species, and antioxidant defenses [9, 10]. Most important to this study is that many types of MSCs are isolated from tissue environments not normally exposed to high levels of oxidative stress, yet when transplanted, they must subsequently function in environments of high, local, or systemic oxidative stress and increased inflammation, such as hypertension, atherosclerosis, angina, thrombosis, Alzheimer's disease, and Parkinson's disease [11–13].

The principle for MSC-based therapies to treat the above diseases is that transplanted MSCs migrate to the sites of inflammation and injured tissue in response to various stimuli including cytokines, chemokines, and growth factors. At these sites, MSCs repair the damaged region in a hostile microenvironment, which can include hypoxia and a milieu of oxidative stress and inflammatory factors. MSCs act either by engrafting and differentiating into tissue-specific cell types or more likely by a paracrine mechanism where they stimulate endogenous stem cells and/or modulate the functions of the innate and adaptive immune cells, such as antigen-presenting cells and lymphocytes [2, 4–7]. MSCs that are unable to resist or succumb to the toxic environment in which they must act will have reduced therapeutic potential [14]. Here, we focus on the effects of oxidative stress on important functions of MSCs.

Recently, we reported that MSCs isolated from the maternal *decidua basalis* tissue (DBMSCs) of human term placenta have unique phenotypic characteristics and ability to prevent inflammation associated with inflammatory diseases [1, 15].

The maternal *decidua basalis* is a major source of oxidized macromolecules that appear in the maternal circulation as a result of pregnancy [16]. DBMSCs in their vascular microenvironment (i.e., their niche) are exposed to elevated levels of inflammation and oxidative stress, which induces resistance in DBMSCs to oxidative stress as previously reported [17]. In addition, our recent studies show that DBMSCs express the antioxidant enzyme aldehyde dehydrogenase 1 (ALDH1) and are more resistant to oxidative stress than the chorionic villus MSCs, which are derived from fetal tissue of the placenta [18–20]. These fetal chorionic MSCs are exposed to the fetal circulation and experience lower levels of inflammation and oxidative stress [18, 19].

Preconditioning MSCs from bone marrow (BMMSCs) and other sources by exposure to hypoxic and oxidative stress-inducing conditions improves many of their stem cell characteristics [21]. Little is known about the properties of preconditioned DBMSCs. In this study, we examined the functional responses of DBMSCs to oxidative stress conditioning. We exposed DBMSCs to various doses of hydrogen peroxide (H_2_O_2_), and their functional properties were evaluated. We found that DBMSCs survive the harsh environment provided by varying doses of H_2_O_2_, and that preconditioning of DBMSCs with H_2_O_2_ enhanced their proliferation, clonogenic ability, adhesion, and migration. In addition, DBMSCs regardless of their H_2_O_2_ treatment showed antiangiogenic activity on endothelial cells. Preconditioning of DBMSC by H_2_O_2_ resulted in enhanced expression of genes that induce the functions of cells. In addition, preconditioned DBMSCs showed reduced expression of genes with antiproliferative and apoptotic activities. Treatment with H_2_O_2_ reduced DBMSC expression of IL-1*β*, an inflammatory cytokine, but had no effects on DBMSC expression of other immune molecules examined in this study and are known for their proliferative, adhesive, and migration properties. These data indicate that preconditioned DBMSCs can function in the hostile, toxic, oxidative, and inflammatory environment associated with many diseases. Preconditioned DBMSCs may increase the likelihood of a successful outcome in stem cell transplantation, for diseases associated with oxidative and other stress.

## 2. Material and Methods

### 2.1. Ethics and Collection of Human Tissues

The ethical research board at King Abdullah International Medical Research Center, Saudi Arabia, approved this study with reference number IRBC/246/13. After consenting normal pregnant women (38–40 weeks of gestation), tissues (placentae and umbilical cords) were collected after vaginal delivery and were then used and processed immediately.

### 2.2. Isolation and Culture of DBMSCs

DBMSCs were isolated from the maternal tissue of human term placenta specifically from the *decidua basalis* region, as previously described [1]. Briefly, tissues (10 grams) were dissected from the placenta and extensively washed with sterile phosphate-buffered saline (PBS, pH 7.4). The tissue was then minced and digested using a PBS solution containing 0.3% collagenase type I (Life Technologies, Grand Island, USA), 271 U/mL DNase I (Life Technologies), and antibiotics (100 *μ*g/mL streptomycin and 100 U/mL penicillin), as previously described [1]. After filtering the mixture with a 100 *μ*m nylon filter (Becton Dickinson, New Jersey, USA), the cell suspension was centrifuged to collect the cell pellet, and red blood cells were then removed using a lysis buffer (catalogue number sc-3621, Santa Cruz, California, USA), as previously described [1]. Cells were then collected and cultured in a complete DBMSC culture medium (Dulbecco's modified Eagle's medium F-12 (DMEM/F-12), 10% MSC certified fetal bovine serum (MSC-FBS) (Life Technologies), and antibiotics as described above) at 37°C in a cell culture incubator (a humidified atmosphere containing 5% CO_2_ and 95% air). At 75% confluency, cells were harvested with a detachment solution (TrypLE express, Life Technologies) and characterized by flow cytometry using MSC and hematopoietic markers (Table 1), as previously described [1]. DBMSCs (passage 3) were differentiated into adipocytes, chondrocytes, and osteocytes, as previously described [1]. DBMSCs were isolated from placentae collected from pregnancies with male births. Prior to the use of DBMSCs in subsequent experiments, DBMSCs were examined for the expression of SRT gene by the real-time polymerase chain reaction (RT-PCR) to exclude their contamination by fetal-derived cells as we previously published [1]. DBMSCs (passage 3) from thirty placentae were used in this study.

### 2.3. Isolation and Culture of HUVEC

HUVEC were isolated from umbilical cord veins, as previously described [22]. Cells were cultured in a complete HUVEC growth medium (catalogue number ATCC® PCS-100-041™, ATCC, USA) at 37°C in a cell culture incubator. At 75% confluency, cells were harvested as described above and characterized by flow cytometry using CD31 endothelial cell marker (R&D Systems). HUVEC with purity (>95%) were used in experiments. Cells (passage 3) from five umbilical cords were used in this study.

### 2.4. H_2_O_2_ Treatment

DBMSCs were cultured in a complete DBMSC culture medium and incubated at 37°C in a cell culture incubator. At 75% confluency, nonadherent cells were removed and DBMSCs were then cultured in a complete DBMSC culture medium without hydrogen peroxide or medium containing hydrogen peroxide (H_2_O_2_, 30% (*w*/*w*) solution, Sigma Aldrich, USA) at a final concentration of 50 and 100 *μ*M and incubated at 37°C in a cell culture incubator for 72 hours. Three groups were used: untreated control DBMSCs (group 1, DBMSC); in-treatment DBMSCs, which were cultured in medium containing 50 *μ*M or 100 *μ*M H_2_O_2_ during the functional assays (group 2, in-DBMSC); and DBMSCs preconditioned with H_2_O_2_, which were DBMSCs initially cultured in medium containing 50 *μ*M or 100 *μ*M H_2_O_2_ (group 3, pre-DBMSC). The viability of DBMSCs in all groups was determined by Trypan blue staining and counting cells using a haemocytometer chamber. The treatment of DBMSCs with various concentrations of H_2_O_2_ (1, 5, 25, 50, 100, 200, 400, and 600 *μ*M) during culture was also examined. DBMSCs cultured without H_2_O_2_ were included as negative controls. Experiments were carried out in triplicate using DBMSCs (passage 3) prepared from five individual placentae.

### 2.5. Cell Proliferation Assay Using a Tetrazolium Compound [3-(4, 5-Dimethylthiazol-2-yl)-5-(3-carboxymethoxyphenyl)-2-(4-sulfophenyl)-2H-tetrazolium, Inner Salt] MTS

To examine DBMSC proliferation in response to H_2_O_2_, DBMSCs from the three groups described above (2.4) were examined. Briefly, cells from each group (group 1, DBMSC; group 2, in-DBMSC; and group 3, pre-DBMSC) were seeded at a density of 5 × 10^3^ per well in 96-well tissue culture plates containing complete DBMSC culture medium and incubated for 24 h at 37°C in a cell culture incubator. Cell proliferation was then evaluated using an MTS kit (catalogue number G5421, Promega, Germany) as previously described [1]. The results were presented as means of standard errors obtained from triplicate samples. The proliferation of DBMSCs exposed to various concentrations of (1, 5, 25, 50, 100, 200, 400, and 600 *μ*M) H_2_O_2_ during culture was also examined. MTS solution in medium not exposed to cells was also used as blank. Experiments were carried out in triplicate using DBMSCs (passage 3) prepared from five independent placentae.

### 2.6. Colony-Forming Unit (CFU) Assay

Colony-forming efficiency of DBMSCs was evaluated as previously described [1]. Briefly, pre-DBMSCs (group 3) were harvested as described above, washed with PBS, seeded into six well plates at a density of 100 cells/well in a complete DBMSC culture medium, and then incubated at 37°C in a cell culture incubator. Untreated DBMSCs (group 1) were used as a control. The medium was replaced with fresh medium every 3 days. After 14 days of culture, the medium was removed and the cells were then washed with PBS and fixed with 4% paraformaldehyde in PBS, pH 7.4 at RT for 30 min. After washing cells with PBS, they were stained with 0.1% Crystal Violet (Santa Cruz) at RT for 15 min, rinsed with distilled water, and visualized, and photomicrographs were recorded as described above. Colonies of cell aggregates of ≥50 cells were scored. Experiments were carried out in triplicate and repeated as described above.

### 2.7. Adhesion Assay

A 96-well plate was coated with 50 *μ*L of 10 *μ*g/mL fibronectin (R&D Systems) and control wells with 50 *μ*L of 1% FBS. After incubation overnight at 4°C, the remaining fibronectin and FBS were removed, and the plate was then washed with PBS. Then, 50 *μ*L 1% FBS was added to the fibronectin wells to block the remaining sites in the fibronectin-coated surface to prevent nonspecific binding. After 2 hours of incubation at RT, FBS was discarded and wells were washed with PBS. Pre-DBMSCs (group 3) were harvested and washed with PBS, and 1 × 10^3^ cells were then added to each well. In-DBMSC (group 2) was treated with 50 and 100 *μ*M H_2_O_2_ during the adhesion assay. After 2 hours of incubation at 37°C in a cell culture incubator, the nonadhered cells were removed gently by washing the wells with PBS, and 100 *μ*L of fresh DMEM/F-12 medium was then added to each well. Finally, MTS was performed as described above. Untreated DBMSCs (group 1) were used as a control. Experiments were carried out in triplicate and repeated as described above.

### 2.8. DBMSC Migration Using xCELLigence Real-Time Cell Analyzer

To examine the effect of H_2_O_2_ on DBMSC migration, the three groups of DBMSCs (untreated control DBMSC, in-DBMSC, and pre-DBMSC) were examined using the xCELLigence real-time cell analyzer (RTCA DP; Roche Diagnostics, Mannheim, Germany), as we previously described [22]. CIM migration plates (catalogue number 05665825001, Roche Diagnostics) were used as previously described [22]. These plates consist of upper and lower chambers separated by a porous (pore size: 8 *μ*m) membrane in conjunction with microelectrodes. Treatments with desired concentrations were prepared in a final volume of 160 *μ*L of culture media and loaded in the lower wells of the migration plate. Following the addition of 50 *μ*L prewarmed media to the wells of the upper chamber, the plates were locked in the RTCA DP device, and equilibrium was then obtained as previously described [22]. A measurement step was performed as a background signal, generated by cell-free media. To initiate the experiment, DBMSCs (20 × 10^3^) prepared as described above were added to the upper chamber in 100 *μ*L, and the plates were then incubated as previously described [22]. In the migration experiment, DBMSCs were seeded in the upper chamber in DBMSC serum-free medium while DBMSC medium supplemented with 20% FBS was added to the lower chamber for all DBMSC groups. The impedance value of each well was automatically monitored for 24 hours and expressed as a cell index (CI) value. Migration observed in the presence of 20% FBS and with medium alone served as positive and negative controls, respectively. Experiments were carried out in quadruplicate and repeated as described above.

### 2.9. Tube Formation Assay

Aliquots (100 *μ*L) of Matrigel® Growth Factor Reduced (GFR) Basement (catalogue number 354230, Corning, USA) were plated into individual wells of 96-well tissue culture plates (Becton Dickinson) and allowed to polymerize overnight at 37°C in a cell culture incubator. In-DBMSC and pre-DBMSC (group 2), pre-DBMSC (group 3) and untreated DBMSCs (group 1) prepared as described above were then seeded at a density of 3 × 10^4^ per well in DBMSC culture medium supplemented with 50 ng/mL vascular endothelial growth factor (VEGF, R&D Systems) on the polymerized Matrigel. HUVEC (3 × 10^4^ per well) cultured without DBMSCs were used as a positive control, or the HUVEC were cultured with 50 or 100 *μ*M H_2_O_2_, or with the three groups of DBMSCs. Varying DBMSC: HUVEC ratios (1 : 30, 1 : 6, and 1 : 4) were used. The concentrations of H_2_O_2_ used for this assay had no effect on the viability of HUVEC as we previously published [22]. Following 14 h of incubation, the tube network was formed and was observed under an inverted Nikon ECLIPSE Ti U microscope (Nikon, Japan), and photomicrographs were recorded using a Nikon DS-Qi1 camera and data were analyzed with Software (Nikon, Japan). Experiments were carried out in triplicate and repeated as described above.

### 2.10. RNA Isolation, cDNA Synthesis, and Real-Time Polymerase Chain Reaction (RT-PCR) Analysis

The expression of 84 genes related to oxidative stress (catalogue number 330231 Qiagen, Hilden, Germany) by DBMSCs was determined using QuantiTect Primer Assay (Qiagen) in a real-time polymerase chain reaction (RT-PCR) as previously published [1]. Briefly, total RNA from DBMSCs treated with or without H_2_O_2_ was isolated, and cDNA was then synthesized using FastLane Cell cDNA kit and RT Primer Mix (Qiagen) as previously published [1]. After quantifying mRNA using QuantiTect SYBR Green PCR kit (Qiagen), the real-time PCR reaction was performed in triplicate on the CFX96 real-time PCR detection system (Bio-Rad) as previously published [1]. Then, the data were analyzed using the CFX manager software (Bio-Rad). The results were exported to Microsoft Excel for further analysis. The results were expressed in terms of fold change by calculating the ΔΔ^−2^ values. The relative expression an internal housekeeping gene as a loading control was used as provided in the kit. Each experiment was carried out using DBMSCs (passage 3) prepared from three individual placentae.

### 2.11. Flow Cytometry

DBMSCs (1 × 10^5^) were stained with monoclonal antibodies (Table 1) for 30 min and phenotypically characterized by flow cytometry as previously described [1]. To analyze intracellular expression of molecules, DBMSCs were fixed and permeabilized as previously described [1]. FACS CANTO II (Becton Dickinson) flow cytometer was used to assess the expression of the intracellular and cell surface markers as described previously [1]. Cells stained with FITC or PE-labeled mouse IgG or IgM antibody were served as negative controls.

### 2.12. Statistical Analysis

GraphPad Prism 5 was used to analyze data using the Mann–Whitney *U* and Kruskal-Wallis tests for nonparametric data. Results were considered to be statistically significant if *P* < 0.05.

## 3. Results

### 3.1. Isolation and Characterization of DBMSCs

DBMSCs are isolated from the *decidua basalis* of the maternal tissue of human term placenta. DBMSCs (passage 3) were (>95%) positive for MSC markers and negative for hematopoietic markers (Table 1). This was consistent with our previously published study [1]. These DBMSCs also differentiated into the bone, fat, and cartilage *in vitro* as we previously published [1]. We also confirmed that all isolated DBMSCs are free from contamination by fetal-derived cells as we previously described [1]. Consequently, DBMSCs (passage 3) were used in this study.

### 3.2. H_2_O_2_ Effects on the Proliferative Potential of DBMSCs

**Figure 1:**
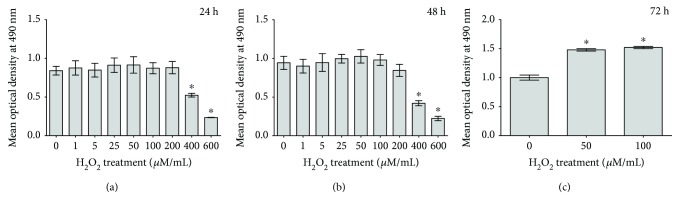
The effect of H_2_O_2_ on DBMSC proliferation determined by the MTS assay. After 24 and 48 h and as compared to untreated DBMSCs, the incubation of DBMSCs with various concentrations of H_2_O_2_ ranging from 1 to 200 *μ*M did not induce a significant change in their proliferation, *P* > 0.05 (a and b) while their incubation with 50 and 100 *μ*M of H_2_O_2_ for 72 h significantly increased their proliferation, ^∗^*P* < 0.05 (c). The incubation of DBMSCs with 400 and 600 *μ*M H_2_O_2_ for the indicated time significantly decreased their proliferation as compared to untreated DBMSCs, *P* < 0.05 (a and b). Experiments were carried out in triplicate using DBMSCs (passage 3) prepared from five individual placentae. Bars represent standard errors.

To evaluate spatial and temporal response of DBMSCs to H_2_O_2_, the cells were cultured in DBMSC culture medium containing H_2_O_2_ or control medium. The proliferation potential was determined using the MTS assay. After 24 and 48 h of treatment with 1, 5, 25, 50, 100, and 200 *μ*M H_2_O_2_, DBMSC proliferation did not change significantly (*P* > 0.05) as compared to untreated DBMSCs (Figures 1(a) and 1(b)) while significant increase (*P* < 0.05) in DBMSC proliferation was observed when cells treated with 50 and 100 *μ*M of H_2_O_2_ for 72 h (Figure 1(c)). Treatment with 400 and 600 *μ*M H_2_O_2_ for the indicated time significantly decreased DBMSC proliferation as compared to untreated DBMSCs, *P* < 0.05 (Figures 1(a) and 1(b)). The viability of DBMSC treated with 1, 5, 25, 50, 100, and 200 *μ*M H_2_O_2_ for all time points was 95%. In contrast, the exposure of DBMSC to 400 and 600 *μ*M H_2_O_2_ for 72 h decreased their viability up to 60% ± 10% and 45% ± 9%, respectively.

Based on the results obtained above, the exposure time of 72 h and two concentrations of H_2_O_2_ (50 and 100 *μ*M) were selected to evaluate the effect of H_2_O_2_ on the functions (clonogenic, adhesion, migration, and tube network formation) of DBMSCs. These concentrations were also selected, because they were not toxic on HUVEC, which were used in the tube formation assay, as previously published [22].

**Figure 2:**
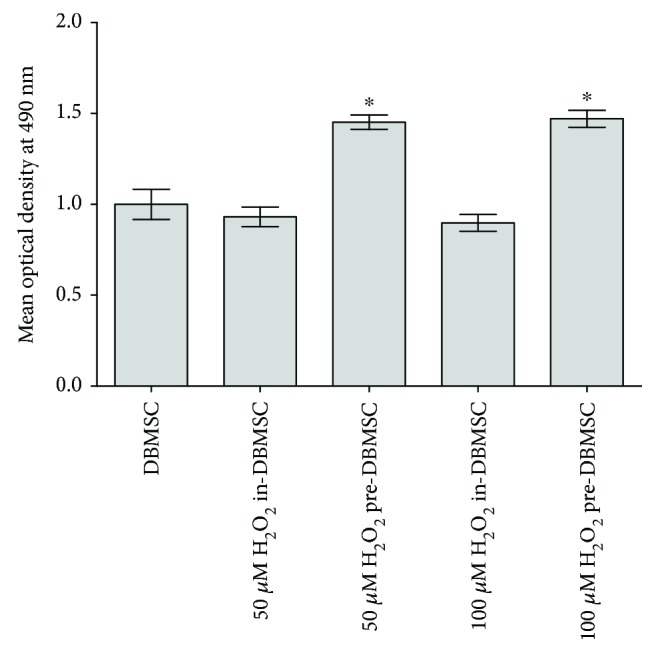
The effect of H_2_O_2_ on DBMSC proliferation determined by the MTS assay. After 24 h, DBMSC (in-DBMSC) proliferation in the presence of 50 and 100 *μ*M H_2_O_2_ did not change significantly (*P* > 0.05) as compared to untreated DBMSCs. The proliferation of DBMSC preconditioned (pre-DBMSC) with 50 and 100 *μ*M H_2_O_2_ significantly increased (^∗^*P* < 0.05) as compared to DBMSC or DBMSC cultured (in-DBMSC) in 50 and 100 *μ*M H_2_O_2_. Experiments were carried out in triplicate using DBMSCs (passage 3) prepared from five individual placentae. Bars represent standard errors.

DBMSC proliferation in presence of 50 and 100 *μ*M H_2_O_2_ for 24 h (in-DBMSC) did not change significantly as compared to untreated DBMSCs (*P* > 0.05), while the proliferation of DBMSCs preconditioned with 50 or 100 *μ*M H_2_O_2_ (pre-DBMSC) for 72 h increased significantly as compared to in-DBMSC and untreated controls (*P* < 0.05) (Figure 2). The effect of H_2_O_2_ on DBMSC proliferation was maintained with increasing time in culture (up to 72 h), and subsequently, the culture time used in this study was 72 h.

### 3.3. Colony-Forming Unit Assay

**Figure 3:**
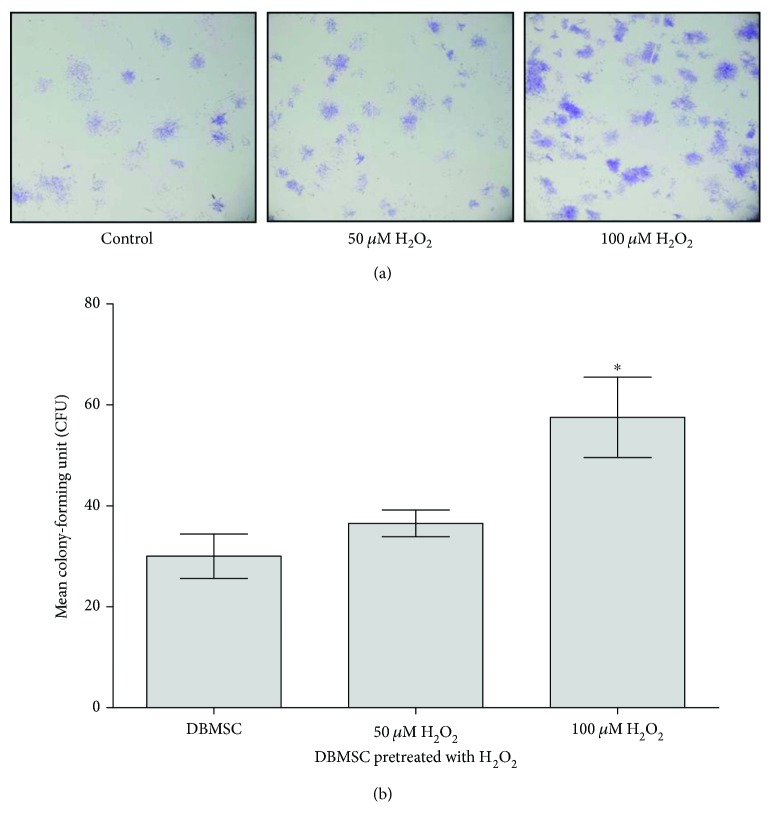
The effect of H_2_O_2_ on colony formation ability of DBMSCs. (a) After 14 h in culture photomicrographs showing representative examples of a colony forming unit of DBMSCs preconditioned (Pre-DBMSC) with 50 and 100 *μ*M H_2_O_2_ or untreated DBMSCs. (b) As compared to control DBMSC, the number of colonies of DBMSC preconditioned (Pre-DBMSC) with 100 *μ*M H_2_O_2_ significantly increased (^∗^*P* < 0.05) while the number of colonies of DBMSC preconditioned (Pre-DBMSC) with 50 *μ*M H_2_O_2_ increased, but not significantly, *P* > 0.05. Experiments were carried out in triplicate using DBMSCs (Passages 3) prepared from five individual placentae. Bars represent standard errors.

DBMSCs preconditioned (pre-DBMSC) with H_2_O_2_ for 72 h were clonogenic as shown by CFU assay (Figures 3(a) and 3(b)). Clusters of ≥50 cells were counted as colonies. Seeding DBMSC preconditioned (pre-DBMSC) with 50 and 100 *μ*M H_2_O_2_ at density of 100 cells per well resulted in 36.50 ± 5.32 and 57.50 ± 15.93 colonies, respectively, while the seeding of untreated DBMSCs resulted in 30 ± 8.79 colonies. The results showed that seeding DBMSC preconditioned (pre-DBMSC) with 100 *μ*m H_2_O_2_ significantly increased colony formation as compared to DBMSC preconditioned (pre-DBMSC) with 50 *μ*m H_2_O_2_ and untreated controls, *P* < 0.05 (Figure 3(b)).

### 3.4. DBMSC Adhesion Is Increased by H_2_O_2_ Preconditioning

**Figure 4:**
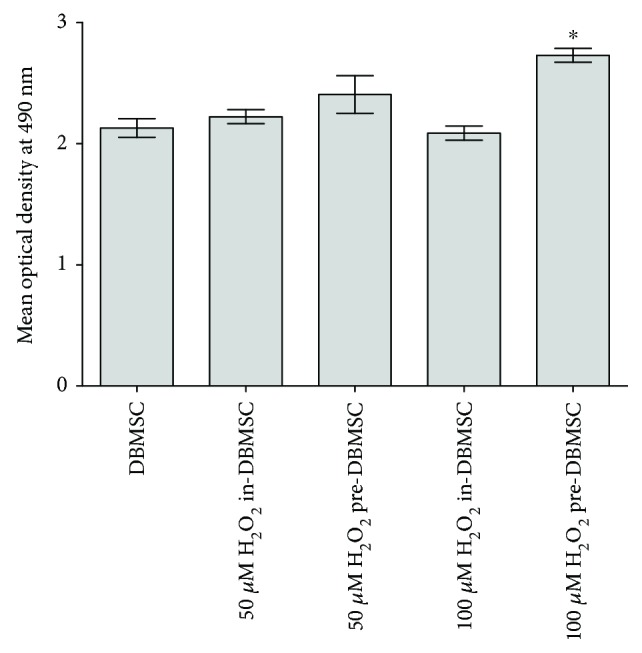
The effect of H_2_O_2_ on the adhesion of DBMSCs measured by the MTS assay. After 2 h and as compared to untreated DBMSC, in-DBMSC adhesion in the presence of 50 and 100 *μ*M H_2_O_2_ was not significantly changed, *P* > 0.05. The adhesion of DBMSC preconditioned (pre-DBMSC) with 50 *μ*M H_2_O_2_ did not change significantly (*P* > 0.05) as compared to in-DBMSC cultured in 50 *μ*M H_2_O_2_ while the adhesion of DBMSC preconditioned (pre-DBMSC) with 100 *μ*M H_2_O_2_ significantly increased (^∗^*P* < 0.05) as compared to in-DBMSC treated with 100 *μ*M H_2_O_2_ and untreated DBMSC. Experiments were carried out in triplicate using DBMSCs (passage 3) prepared from five individual placentae. Bars represent standard errors.

To evaluate the effect of H_2_O_2_ on the adhesion of DBMSCs, DBMSC adhesion to fibronectin-coated surface was evaluated using MTS assay. DBMSC preconditioned (pre-DBMSC) with 100 *μ*M H_2_O_2_ for 72 h showed significantly increased adhesion to the fibronectin-coated surface as compared to untreated DBMSCs, *P* < 0.05 (Figure 4). In contrast, DBMSC preconditioned (pre-DBMSC) with 50 *μ*M H_2_O_2_ for 72 h showed increased adhesion, but this was not statistically significant as compared to untreated DBMSCs, *P* > 0.05 (Figure 4). DBMSC treated (in-DBMSC) with either 50 or 100 *μ*M showed no significant increase in adhesion as compared to untreated DBMSCs, *P* > 0.05 (Figure 4).

### 3.5. DBMSC Migration Is Increased by H_2_O_2_ Preconditioning

**Figure 5:**
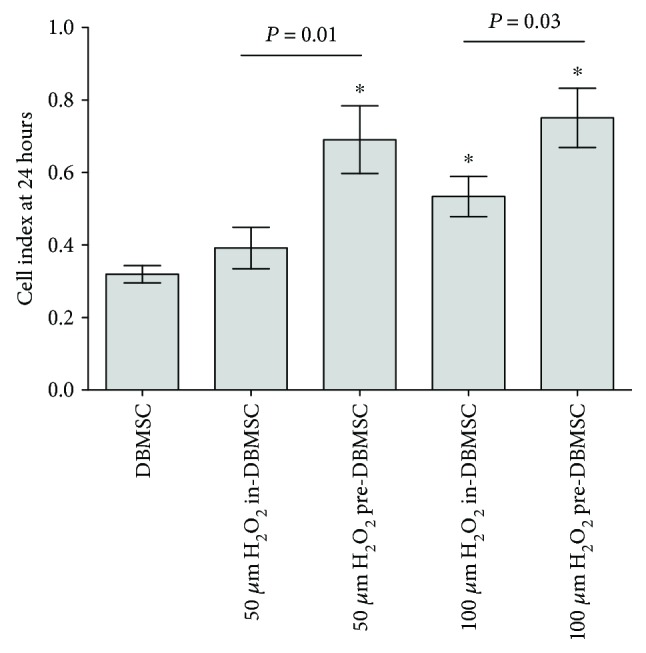
The effect of H_2_O_2_ on DBMSC migration measured using the xCELLigence real-time cell analyzer. After 24 h and as compared to untreated DBMSC, the migration of DBMSC treated (in-DBMSC) with 50 *μ*M H_2_O_2_ changed, but not significantly, *P* > 0.05. In contrast, the migration of DBMSC treated (in-DBMSC) with 100 *μ*M H_2_O_2_ significantly increased as compared to untreated DBMSC, ^∗^*P* < 0.05. The migration of DBMSC preconditioned (pre-DBMSC) with 50 and 100 *μ*M H_2_O_2_ significantly increased as compared to in-DBMSC treated with 50 and 100 *μ*M H_2_O_2_ or to untreated DBMSC, ^∗^*P* < 0.05. Experiments were carried out in triplicate DBMSCs (passage 3) prepared from five individual placentae. Bars represent standard errors.

To evaluate the effect of oxidative stress preconditioning on the migration potential of DBMSCs, DBMSCs from groups 1, 2, and 3 were used in a migration assay. The migration of DBMSCs was evaluated in response to FBS as a chemotactic factor added into the lower chamber of a migration plate and was measured using the xCELLigence real-time cell analyzer. After 24 h, the migration of DBMSC treated (in-DBMSC) with 50 *μ*M H_2_O_2_ changed but was not statistically significant as compared to untreated DBMSCs, *P* > 0.05 (Figure 5). In contrast, the migration of DBMSC treated (in-DBMSC) with 100 *μ*M H_2_O_2_ significantly increased as compared to untreated DBMSCs, *P* < 0.05 (Figure 5). Similarly, the migration of DBMSC preconditioned (pre-DBMSC) with 50 and 100 *μ*M H_2_O_2_ significantly increased after 24 h as compared to untreated DBMSCs or to DBMSC treated (in-DBMSC) with 50 and 100 *μ*M H_2_O_2_*P* < 0.05 (Figure 5).

### 3.6. DBMSC Tube Network Formation In Vitro

**Figure 6:**
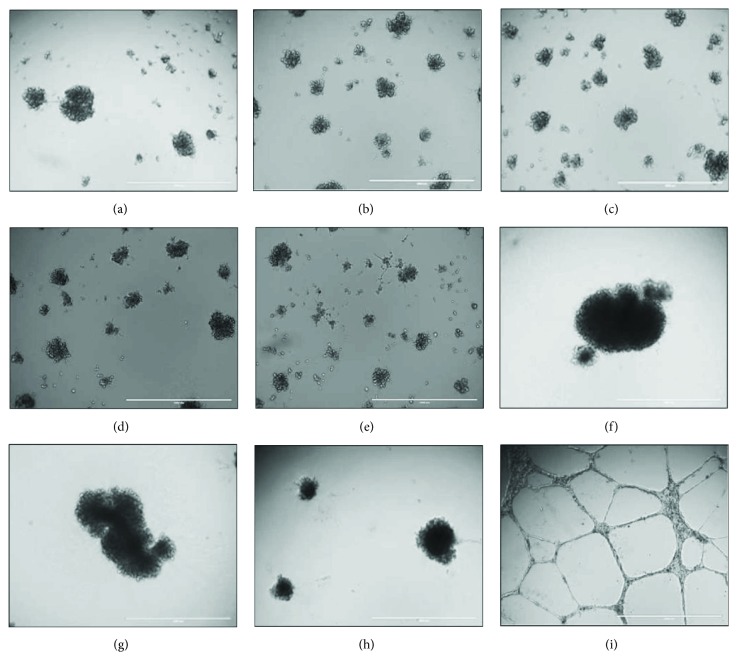
The ability of DBMSCs to form tube networks and their inhibitory effects on HUVEC formation of tube networks. After 14 h, H_2_O_2_ untreated DBMSCs (a) and DBMSC pretreated with 50 *μ*M H_2_O_2_ (b) and 100 *μ*M H_2_O_2_ (c); DBMSCs cultured in 50 *μ*M H_2_O_2_ (d) or 100 *μ*M H_2_O_2_ were unable to form tube networks. The addition of H_2_O_2_ untreated DBMSCs (f) and DBMSC pretreated with 50 *μ*M H_2_O_2_ (g) and 100 *μ*M H_2_O_2_ (h) to HUVEC completely inhibited HUVEC formation of tube networks as compared to H_2_O_2_-untreated HUVEC (i). Experiments were carried out in triplicate DBMSCs (passage 3) prepared from five individual placentae.

To evaluate the ability of DBMSCs to form tube network *in vitro*, DBMSCs from groups 1, 2, and 3 were seeded on a Matrigel-coated surface. After 14 h, untreated DBMSCs (Figure 6(a)) and DBMSC preconditioned (pre-DBMSC) with 50 *μ*M H_2_O_2_ (Figure 6(b)) and 100 *μ*M H_2_O_2_ (Figure 6(c)) were unable to form tube networks. Similarly, DBMSCs treated (in-DBMSC) with either 50 or 100 *μ*M were also unable to form tube networks (Figures 6(d) and 6(e)). In addition, the addition of untreated DBMSCs (Figure 6(f)) and DBMSC preconditioned (pre-DBMSC) with 50 *μ*M H_2_O_2_ (Figure 6(g)) and 100 *μ*M H_2_O_2_ (Figure 6(h)) to HUVEC culture completely blocked HUVEC formation of tube networks as compared to H_2_O_2_-untreated HUVEC (Figure 6(i)). The addition of 50 *μ*M or 100 *μ*M H_2_O_2_ to HUVEC culture did not affect the ability of HUVEC to form tube networks as previously reported [23]. Finally, the addition of DBMSCs to HUVEC in the presence of 50 *μ*M or 100 *μ*M H_2_O_2_ completely blocked HUVEC formation of tube networks as compared to untreated HUVEC.

### 3.7. H_2_O_2_ Preconditioning Enhances the Expression of Prosurvival Molecules in DBMSCs

Differential expression of oxidative stress genes in DBMSCs, before and after H_2_O_2_ preconditioning, was analyzed and assessed using the real-time PCR assay. RNA isolated from untreated and DBMSC preconditioned with 50 *μ*M and 100 *μ*M H_2_O_2_ for 72 h was transcribed into cDNA and added to the commercially available 96-well plates of RT^2^ Profiler Oxidative Stress kit (Qiagen). Results as shown in Table 2 show that preconditioning with H_2_O_2_ increased (Table 2(a)) and decreased (Table 2(b)) the expression of several oxidative stress-related genes as compared to untreated DBMSCs. Modulation in the expression profiles was dose dependent and was statistically significant, *P* < 0.05.

### 3.8. DBMSC Expression of Inflammatory Molecules

**Figure 7:**
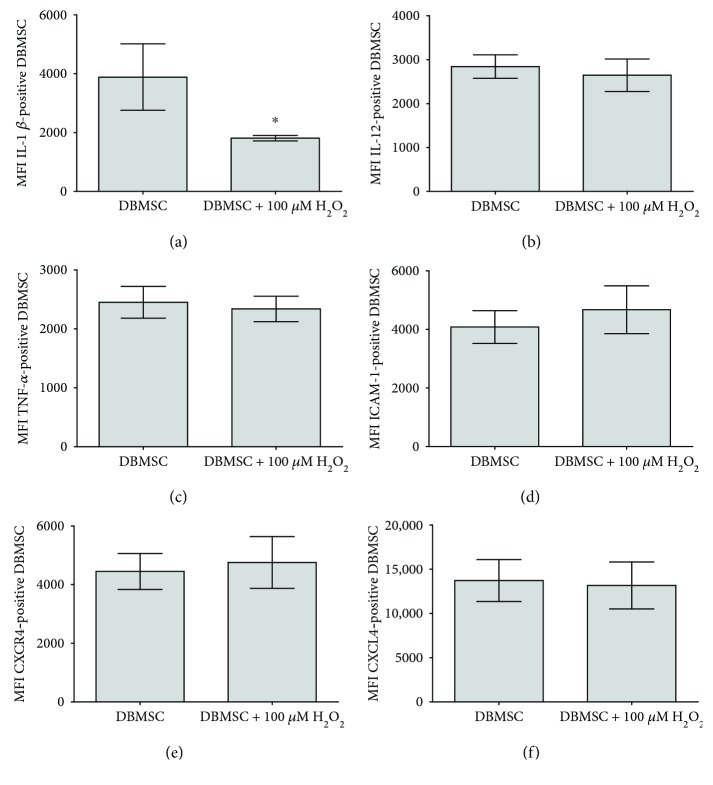
Flow cytometric analysis of DBMSC expression of immune markers. After 24 h of culture with 100 *μ*M H_2_O_2_ and as compared with untreated control DBMSC, DBMSC expression of IL-1*β* was significantly reduced (^∗^*P* < 0.05) while the expression of IL-12, TNF-*α*, ICAM-1, CXCR4, and CXCL4 by DBMSCs (b–e, resp.) was not significantly changed (*P* > 0.05). The levels of the expression of immune markers in DBMSCs were recorded as median fluorescence intensity. Experiments were conducted in triplicate using DBMSCs (passage 3) prepared from five placentae. Bars represent standard errors.

The modulatory effects of H_2_O_2_ on DBMSCs were evaluated by studying the expression of a variety of immune molecules using flow cytometry, and the expression was recorded as median fluorescence intensity. After 24 hours of culture with 100 *μ*M H_2_O_2_, DBMSC expression of IL-1*β* was significantly reduced as compared with untreated DBMSCs, *P* < 0.05 (Figure 7(a)) while the expression of IL-12, TNF-*α*, ICAM-1, CXCR4, and CXCl-4 by DBMSCs was not significantly affected, *P* > 0.05 (Figures 7(b)–7(e), resp.). The effect of H_2_O_2_ on DBMSC expression of immune molecules was maintained with increasing time in culture (up to 72 h).

## 4. Discussion

We previously reported that DBMSCs have the characteristics and functions that make them potentially useful for therapies to treat inflammatory diseases, such as atherosclerosis [1, 11–13]. Inflammatory diseases are characterized by high levels of inflammatory and oxidative stress mediators that induce damage in tissues. In this situation, MSCs must repair the damaged tissues in a toxic environment. Therefore, for successful utilization of DBMSCs in transplantation, it is imperative to study their functional responses to disease environments. In this study, the functions of DBMSCs in response to H_2_O_2_ were studied. First, we examined the effect of different H_2_O_2_ concentrations on the survival of DBMSCs. We report that DBMSCs are resistant to high levels of H_2_O_2_ for a longer period in culture. At levels of 400 and 600 *μ*M H_2_O_2_, the viability of DBMSCs after 72 h of exposure significantly reduced. In contrast, the exposure of MSCs derived from the chorionic villi of human placentae (pMSCs) to 400 and 600 *μ*M H_2_O_2_ induced death after 24 h, as previously reported [22] while the exposure of bone marrow-derived MSCs (BMMSCs) to a concentration of H_2_O_2_ lower than 400 *μ*M induced death in these cells suggesting that they are more sensitive to oxidative stress [24, 25]. The ability of DBMSCs to remain viable after treatment with high concentration of H_2_O_2_ as compared to pMSCs and BMMSCs could be attributable to their niche of origin. DBMSCs are normally found in a vascular niche around maternal vessels in the decidua and thus are continuously open to the high levels of inflammation and other stressful conditions throughout normal human pregnancy [26, 27]. In contrast, pMSCs in the chorionic vascular niche are exposed to the fetal circulation and exposed relatively to a lower levels of inflammation and oxidative stress mediators during normal pregnancy [18, 19]. Similarly, BMMSCs are normally exposed to low levels of oxidative stress in their niche and only experience increased oxidative stress during injury or disease [28]. The ability of DBMSCs to resist H_2_O_2_-induced oxidative stress in cell culture was supported by their ability to proliferate under a continuous exposure to H_2_O_2_ for up to 72 h. Interestingly, the proliferative potential of DBMSCs increased following their preconditioning with H_2_O_2_.

We also found that the preconditioning of DBMSCs with H_2_O_2_ enhanced the clonogenicity of these cells, and this was enhanced with a higher concentration of H_2_O_2_. The ability of DBMSCs to retain their clonogenic potential in extended culture further supports their ability to proliferate under oxidative stress, while maintaining their stemness [29, 30]. This is the first study to demonstrate that preconditioning of DBMSCs with H_2_O_2_ enhances their proliferative potential. Consistent with our findings, preconditioning of BMMSCs with other oxidative stress agents, such as hypoxia, also increases their clonogenic potential [31]. A further study confirmed the beneficial effect of hypoxia on the clonogenicity and proliferative potential of adipose tissue-derived MSCs [32, 33].

DBMSCs express and secrete a unique combination of molecules involved in many important cellular functions, such as proliferation [1]. For example, DBMSCs express IL1*β*, IL-12, and TNF-*α*, which have proliferative functions [34–36]. Following preconditioning with H_2_O_2_, DBMSC expression of IL-1*β* significantly reduced, whereas there was no effect of the expression of IL-12 and TNF-*α*. These data suggest that other molecules could mediate the enhanced effect of H_2_O_2_ on the proliferative potential of DBMSCs, as previously reported for MSCs from cord tissue [37]. Preconditioning of these MSCs with H_2_O_2_ enhanced cord tissue MSC expression of VEGF and IL-6 [37], which were shown in other studies to mediate the proliferative function of BMMSCs [38, 39]. Since DBMSCs express these molecules [1], a future study is essential to determine the role of them and other molecules, as well as the mechanisms underlying the induced proliferative potential of DBMSCs in response to H_2_O_2_.

These data indicate that the preconditioning of DBMSCs with H_2_O_2_ improves their proliferation and clonogenicity. This phenomenon of preconditioning-induced protection was previously reported [40]. It was shown that the exposure of myocardial stem cells to a sublethal ischemic environmental condition induced heart resistance to severe ischemia in animals [40]. Similarly, the transplantation of H_2_O_2_-preconditioned cardiac stem cells into animals with myocardial infraction improved heart function [41]. In addition, preconditioning of cardiac stem cells with H_2_O_2_ protected them from apoptosis and improved their differentiation potential [41]. Moreover, other studies reported that the functions (survival, migration, chemotaxis, engraftment, and differentiation) of MSCs increased after their exposure to other stress conditions, such as hypoxic and serum deprivation conditions [42].

In this study, preconditioning of DBMSCs with H_2_O_2_ enhanced their adhesion. This is consistent with a previous study where preconditioning of hematopoietic stem cells with H_2_O_2_ enhanced their adhesion both *in vitro* and *in vivo* [43]. Enhancing adhesion is important since this biological process is the first essential step for a successful engraftment of stem cells [44, 45]. Migration is another important functional activity of MSCs, which must take place within a disease environment of increased inflammation and oxidative stress [44, 45]. We found that H_2_O_2_ had a stimulatory effect on DBMSC migration, and that preconditioning of DBMSCs with H_2_O_2_ enhanced their ability to migrate. This result is consistent with a previous study, which reported that the preconditioning of BMMSCs with H_2_O_2_ promotes their migration potential [25].

In this study, we identified the molecular changes occurring in DBMSCs post preconditioning with H_2_O_2_. We found that H_2_O_2_ upregulated the expression of a number of genes (Table 2(a)) that are known for their antioxidative functions and proproliferative and prosurvival activities in different types of cancers, such as esophageal, colon, stomach, lung, and glioblastomas [46–52]. In addition, these genes can protect the neural cells from death by apoptosis and have an antiapoptotic roles in ovarian follicles [50, 53–58]. We also found that H_2_O_2_ downregulated the expression of a number of genes (Table 2(a)) that have antiproliferative and apoptotic activities [59–67]_._ Modulations of the expression of these genes explain the enhanced proliferation and migratory potential of preconditioned DBMSCs [59–67].

In this study, we also found that DBMSCs were unable to form tube networks *in vitro*, although they express and secrete several proangiogenic factors, such as IL-6, IL1*β*, MCSF, GMCSF, and VEGF [1]. In addition, DBMSCs inhibited endothelial cell formation of network tubes *in vitro* regardless of their H_2_O_2_ treatment. These results indicate that DBMSCs have antiangiogenic activity on endothelial cells. Importantly, oxidative stress has no modulatory effect on this inhibitory function of DBMSCs on endothelial cells. Enhanced angiogenesis is hallmark of many inflammatory diseases, which is associated with increased level of inflammation and oxidative stress. Therefore, our data suggest that DBMSCs could be useful in treating inflammatory diseases by inhibiting angiogenesis. We previously reported that DBMSCs express and secrete several antiangiogenic factors, such as IL1ra, IL-10, and IFN-*γ* [1], which have antiangiogenic activity [67–70], and therefore, they may be responsible for mediating the antiangiogenic activity of DBMSCs on endothelial cells. However, the mechanism by which DBMSCs inhibit angiogenesis should be addressed in future studies.

## 5. Conclusion

This study shows for the first time the beneficial effects of H_2_O_2_ preconditioning on DBMSC proliferation, clonogenicity, adhesion, and migration. Preconditioning DBMSCs may enhance their therapeutic potential by increasing their engraftment efficiency. Moreover, the antiangiogenic properties of DBMSCs may counteract the increased angiogenesis associated with many inflammatory diseases. Future *in vitro* studies are necessary to elucidate the mechanism underlying the enhanced functional properties of H_2_O_2_-preconditioned DBMSCs and to determine the effects of other preconditioning regimes on DBMSC functions. Finally, the effects of preconditioned DBMSCs following transplantation into in an animal model of inflammatory disease will provide supporting evidence of enhanced therapeutic potential.

## Figures and Tables

**Table 1:** Antibodies used in this study.

MSC-positive markers	Hematopoietic markers	Immune markers
CD44	CD14	IL-1*β*
CD90	CD19	IL-12
CD105	CD40	TNF-*α*
CD146	CD45	ICAM-1
CD166	CD80	CXCL4
HLA-ABC	CD83	CXCR4
	CD86	
	HLA-DR	

**Table:** (a) Upregulated genes in DBMSCs after preconditioning

Gene	Functions
ATOX1	Prosurvival, plays a significant role in carcinogenesis [52]
CYGB	Protective function during oxidative stress [45]
DHCR24	Proproliferative, protects neuronal cells from apoptotic cell death [53]
DUOX1	Proproliferative, plays a role in cellular migration [54]
DUOX2	Proproliferative, plays a role in esophageal, stomach, and colon cancer progression [48]
DUSP1	Involved in proliferation and differentiation in various human cancers [55]
FOXM1	Execution of mitosis, cell cycle progression, and other signal pathways leading to tumorigenesis [57]
GCLM	Plays antiapoptotic roles in ovarian follicles [56]
MBL2	Prosurvival, used for protection against oxidative stress in neurons [46]
OXR1	Plays a role in the etiology of glioma [51]
OXSR1	Prosurvival, protection against oxidative stress damage [47]
PRDX4	Oncogenic, highly expressed in a majority of human GBMs as well as in a mouse model of GBM [50]
TXNRD1	Overexpressed in many malignancies including lung cancer [49]

**Table:** (b) Downregulated genes under preconditioning

Gene	Functions
APOE1	Leads to suppression of T cell proliferation [58]
BNIP3	Role in cell death [59]
EPX	Tumor suppressor [60]
FTH1	FTH1 can inhibit Daxx-mediated apoptosis [61]
GPX1	Deficiency causes increased migration and invasion of cells [62]
GPX3	Frequently deleted in prostate cancer [63]
PDLIM1	Deficiency causes increase in proinflammatory cytokines [64]
PRDX2	Silenced in Hodgkin and Reed-Sternberg cells of classical Hodgkin lymphoma [65]
PRNP	Role in cell proliferation, differentiation, death, and survival [66]
